# The Effectiveness of Metronidazole as a Localized Drug Delivery System in the Treatment of Periodontal Diseases: A Narrative Review

**DOI:** 10.7759/cureus.80547

**Published:** 2025-03-13

**Authors:** Utsav H Gandhi, Shruti D Vyas, Vaishnavi Mane, Shirishkumar N Patel, Hiren H Patadiya, Santosh Kumar, Mainul Haque

**Affiliations:** 1 Department of Periodontology and Implantology, Karnavati School of Dentistry, Karnavati University, Gandhinagar, IND; 2 Department of Pharmaceutics, Krishna Institute of Pharmacy, Krishna Vishwa Vidyapeeth, Karad, IND; 3 Department of General Dentistry, My Dental Southbridge PLLC, Southbridge, USA; 4 Department of Pharmacology and Therapeutics, National Defence University of Malaysia, Kuala Lumpur, MYS; 5 Department of Research, Karnavati School of Dentistry, Karnavati University, Gandhinagar, IND

**Keywords:** actinohacillus actinomycetemcomitans, alveolar bone resorption, anaerobic medicine, azole, gram-negative microbes, localized drug delivery system, periodontal disease (pd), porphyromonas gingivalis, tannerella forsythia, treponema denticola

## Abstract

Periodontitis is a complex, multifactorial chronic inflammatory condition that impacts the adjacent hard and soft tissues. Microorganisms, especially gram-negative anaerobic pathogens, are a causative factor for periodontal disease. Periodontitis is identified by observing deeper periodontal pockets, clinical attachment loss, and the reduction of alveolar bone, often in conjunction with these indicators. The condition can vary in severity and be classified as mild, moderate, or severe. Scaling and root planing, combined with mechanical debridement, may not adequately reduce the bacterial load; therefore, adding local or systemic antimicrobials is advised as an adjunctive treatment. Commonly utilized local drug delivery agents for patients suffering from periodontitis include tetracycline, metronidazole, minocycline, doxycycline, and chlorhexidine. This system targets the pockets and eliminates the pathogens. Metronidazole is a nitroimidazole compound used commonly against gram-negative anaerobes. Its mechanism lies in four basic steps through which bacterial cell death occurs. A 25% metronidazole gel is used widely in periodontitis patients. The effectiveness of metronidazole as a local drug delivery agent has been evaluated in numerous studies, which have shown improvements in clinical parameters. To achieve favorable clinical outcomes, the non-surgical treatment of peri-implantitis should involve the systemic or local administration of metronidazole. Thus, the role of metronidazole in the emergence of periodontal diseases and its therapeutic uses are investigated in this narrative review.

## Introduction and background

Periodontitis represents an inflammatory pathology that impacts the supportive structures associated with the dentition in which the alveolar bone and the periodontal ligament gradually deteriorate as a result of this condition [1]. The development of plaque and calculus is one of the primary factors contributing to periodontal disease, as it exacerbates inflammation [2]. This process often begins with gingival inflammation that extends into the deepening of periodontal pockets and loss of adjacent bone [1,2]. Periodontal disease may manifest as either localized or generalized, contingent upon the specific teeth affected, with a localized presentation characterized by involvement of less than 30% of the total dentition [3-5].

Destructive pathogens, such as *Porphyromonas gingivalis* [6], *Aggregatibacter actinomycetemcomitans* [7], *Tanerella forsythias* [8], and *Treponema denticola* [9], may cause periodontal diseases [10,11]. Before moving from non-periodontal regions to periodontal crevices, these pathogens can reside in the tonsils, oral mucosa, and tongue surfaces, among other parts of the oral cavity [12]. Periodontal treatment aims to reestablish equilibrium by diminishing the presence of these pathogens and enhancing patient health care through non-surgical techniques, including scaling and root planing, as well as surgical interventions like pocket reduction surgery.

**Figure 1:**
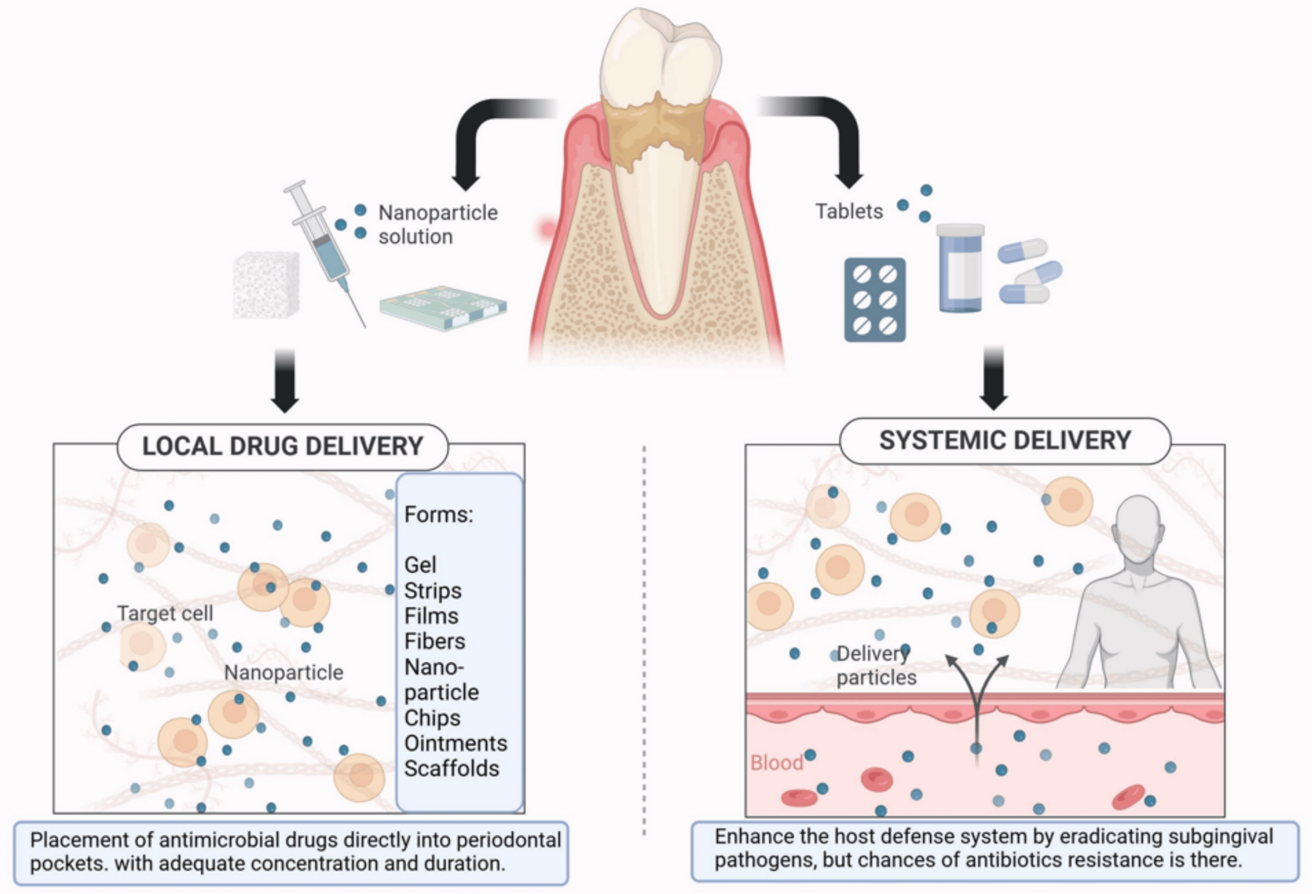
Local vs. systemic drug administration: forms and applications. Notes: The image was created with BioRender's premium version (https://biorender.com/v91s510) with an Agreement license number SS27YBE5I6 [15]. Credit: Utsav Gandhi

Scaling, root planing, and strict home care can help control infection by removing biofilm and supporting periodontal disease management with systemic or local host-modulating agents [13]. Due to the relation of microorganisms with periodontal disease, administering local and/or systemic antimicrobials is recommended to manage periodontal disease to enhance infection control, minimize tissue damage caused by the immune response, and promote optimal healing [14]. Systemic and local drug delivery are applied in various forms to improve the condition of periodontal tissue, as described in Figure 1.

Systemic antibiotics can reach periodontal tissues and effectively target subgingival pathogens in periodontal pockets, which may be difficult to access with traditional mechanical debridement [12,16,17]. Systemically applied antibiotics can increase the chances of antibiotic resistance and adverse drug reactions if used in the long term [18].

Local drug delivery agents within periodontal pockets can potentially suppress or eradicate periodontal pathogens while modulating the surrounding tissues' inflammatory responses [19]. Some of the commonly used drugs are tetracyclines [20], metronidazole [16], chlorhexidine [21], doxycycline [22], minocycline [23], alendronate [24], herbals [25]. These medications are available in multiple forms, including gels, fibers, strips, films, chips, ointments, scaffolds, nanoparticles, and polymers [26-29]. These drugs act at a specific site, remain there for a sufficient time, and help prevent the spread of pathogens [30]. 

Metronidazole, a synthetic compound derived from a 5-nitroimidazole antimicrobial compound [31], acts against gram-negative anaerobic species and exerts bactericidal actions [31-33]. Its efficacy is shown. It is widely used for gingivitis, periodontal diseases, and oral prophylaxis [34,35]. Metronidazole and amoxicillin yield positive results when used with scaling and root planing [36,37].

Metronidazole is a notable pharmacological agent for treating periodontal disease because it is effective against critical microbial pathogens, has a low risk of resistance development, and is economically viable [38,39]. The systemic administration of metronidazole may not effectively reach deep periodontal pockets, whereas local drug delivery ensures a higher concentration at the infection site [40]. This targeted approach enhances clinical outcomes and minimizes systemic side effects [41]. Due to its superior bacterial control and pocket-depth reduction, local metronidazole is more effective than scaling and root planing alone [42].

Problem statement of this study

Periodontal diseases are chronic inflammatory diseases, and their prevalence increases with age [43,44]. The incidence increases significantly among adults aged 30 to 40 [44]. In 2017, Fatimah et al. conducted an epidemiological study that revealed that the highest prevalence of chronic periodontitis was found in older people (82%), followed by adults (73%) and adolescents (59%) [43,45]. Periodontitis is also a reason behind multiple tooth loss and masticatory dysfunction, so it affects the nutrition taken by the patients, increases healthcare costs, leads to socio-economic impact, and hampers the quality of life [44,46]. On the other hand, long-term systemic antibiotic use increases the risk of antibiotic resistance and can have adverse effects [18]. Therefore, the significance of locally delivered drugs needs to be further explored.

Objectives of this narrative review

This paper aims to evaluate and describe the effects of metronidazole on both soft and hard tissues and show how the drug contributes to the development of periodontal diseases. This review also discusses its current clinical applications in localized therapy.

## Review

Materials and methods

Search Strategy

**Figure 2:**
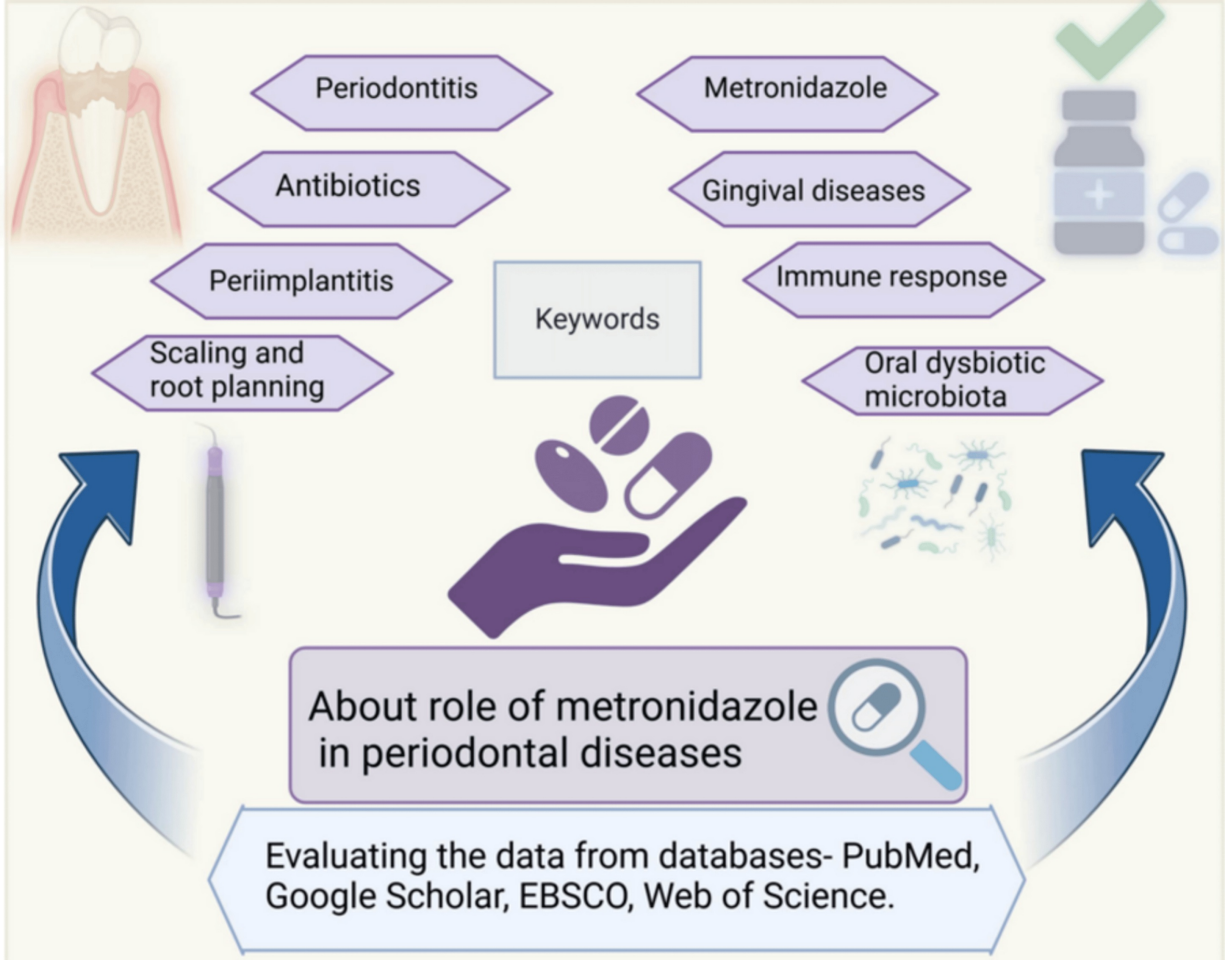
Methodology of the study. Notes: The figure was drawn using the premium version of Biorender (https://biorender.com/e17v396) with agreement license number BM27Y4RAYZ [15]. Credit: Utsav Gandhi

This review analyzed the data from databases (PubMed, Google Scholar, EBSCO, Web of Science, and manual research) in English with study designs such as narrative reviews, systematic reviews, and clinical studies (Figure 2). The keywords utilized in this review are: “Local drug delivery agent,” AND “Periodontitis,” “Metronidazole,” AND “Scaling and root planning,” AND “Gingival disease,” AND “Antibiotic,” “Peri-implantitis,” AND “Oral dysbiotic microbiota.”

Eligibility Criteria

The inclusion criteria encompass studies that examine the effects of metronidazole on periodontal tissue, with a primary focus on the gingiva, bone, and soft tissues, in patients undergoing local drug delivery after scaling and root planning. The exclusion criteria include research published in languages other than English, in vitro research, animal studies, and investigations involving patients with medical complications.

Review of literature

Periodontal diseases are chronic inflammation that causes alveolar bone to be destroyed and periodontal ligaments to deteriorate [1,47-49]. It is considered a major oral health problem worldwide [49]. The risk factors involved in periodontitis are either modifiable or non-modifiable [47,50]. Psychological factors, habits such as smoking, inadequate oral hygiene, certain medications, diabetes mellitus, and microorganisms are considered modifiable elements. In contrast, non-modifiable factors encompass genetic predispositions, host responses, osteoporosis, the aging process, and various systemic diseases [47,50]. Periodontal disease is recognized as a contributing factor to several systemic conditions, such as respiratory diseases, cardiovascular diseases, peripheral arterial diseases, adverse pregnancy outcomes, neurological disorders, chronic kidney disease, preterm low birth weight, and rheumatoid arthritis [47-49,51,52].

Periodontal disease manifests with symptoms such as redness and swelling of gums, bad breath, pain around the teeth and gingiva, and developing periodontal pockets. As the condition progresses, it can result in a gradual detachment of the tooth from the surrounding gums and alveolar bone, ultimately leading to heightened tooth mobility and, if left untreated, tooth loss [49,51]. Periodontitis ranges from mild to severe forms. About 50% of adults worldwide suffer from mild to moderate periodontitis. In contrast, the prevalence of severe periodontitis rises to about 10% during the transition from the third to the fourth decade of life [53].

**Figure 3:**
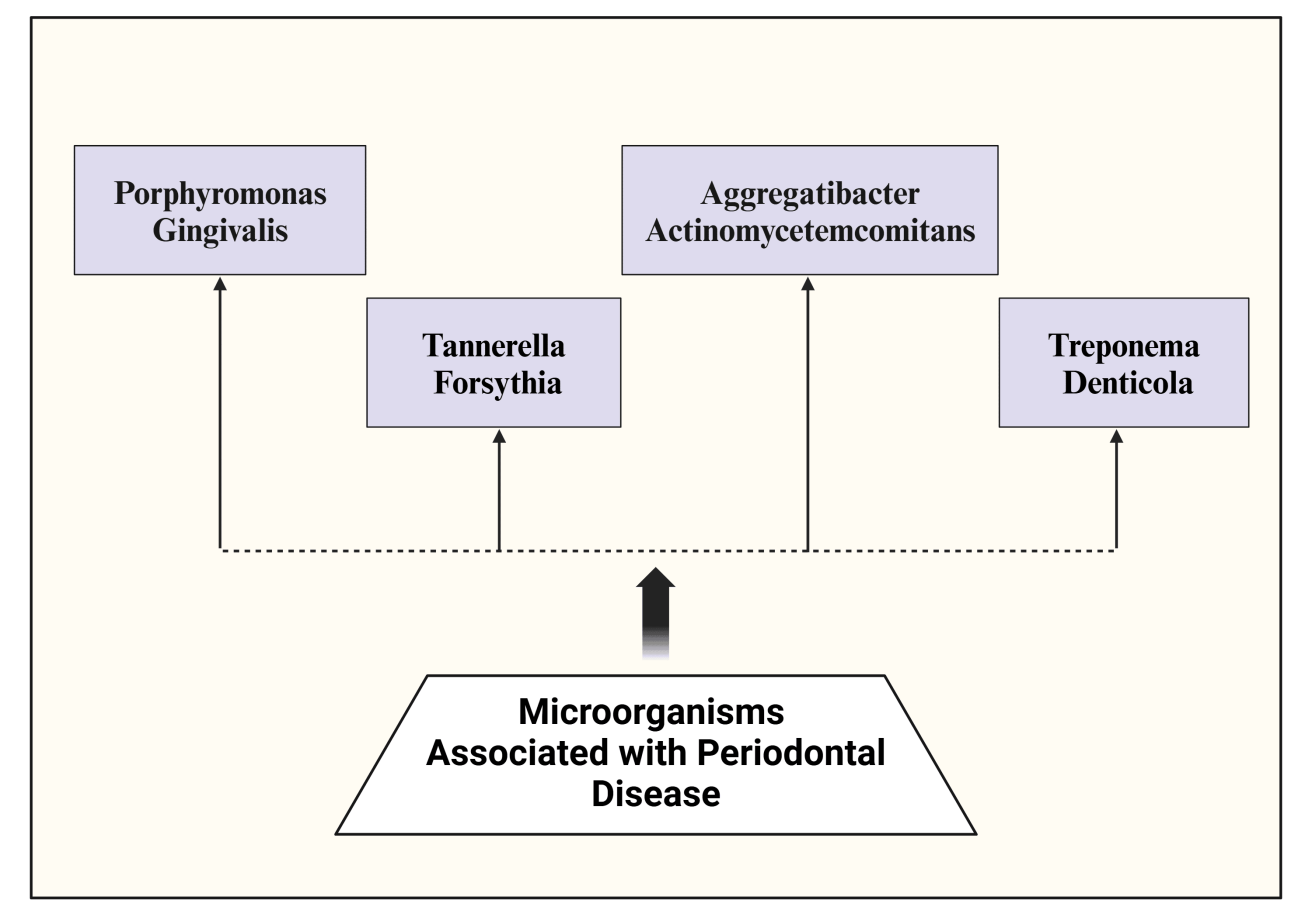
Microorganisms associated with periodontal disease. Notes: The figure was drawn using the premium version of Biorender (https://biorender.com/d11g924) with agreement license number YV27Z3X9II [15]. Credit: Utsav Gandhi

The disease starts with plaque buildup around the teeth, which forms microbial biofilms with bacteria and leads to localized gingiva inflammation [54]. Gram-positive oral bacteria like *Peptostreptococcus*, *Lactobacillus*, and *Streptococcus* are primarily replaced by gram-negative anaerobic bacteria in patients with periodontal disease [55]. *P. gingivalis*, *A. actinomycetemcomitans*, *T. forsythia*, and *T. denticola *are the primary periodontal pathogens (Figure 3) [56,57]. 

Both non-surgical and surgical methods are used to treat periodontitis. Non-surgical techniques like scaling, root planing, and localized medication administration are used for mild to moderate periodontitis. In contrast, more severe instances necessitate surgical intervention after non-surgical treatments. Anaerobic bacteria are essential in the progression of periodontal diseases [16]. Scaling and root planing is the accepted course of treatment, but it may not effectively eliminate all bacteria from the deeper areas of the periodontal pockets. To address this limitation, local or systemic antimicrobial therapy can reach and target the bacteria residing in these deeper regions, enhancing the overall effectiveness of periodontal treatment [58]. After Phase-I therapy, conventional mechanical therapy should be combined with systemic antibiotics to gain clinical improvement [59].

Local Drug Delivery System

This system comes in two variations: non-biodegradable and biodegradable [49]. Non-biodegradable options, like implants, necessitate removal once the drug has been dispensed, which can be a lengthy process and may cause harm to the surrounding new tissue [60]. Biodegradable systems, conversely, break down naturally when exposed to gingival fluid. Fibers, powders, strips, pastes, gels, and ointments are biodegradable formats [61,62]. Local drug delivery is classified into two types: sustained release, lasting less than 24 hours, and controlled release, extending beyond 24 hours [62]. Drug delivery systems facilitate the regulated and extended release of medication at designated locations, ensuring that target agents are effectively administered. They also lessen the amount of medication required and the frequency of administration [63-65]. Beyond dentistry, this drug delivery system is also utilized in cardiology [66], ophthalmology [67], and tumor therapy [68,69]. LDD targets the periodontal pocket [70,71], enabling the simultaneous administration of multiple drugs in a non-invasive manner [65,72,73]. 

In 1985, Goodson outlined a proficient drug delivery system for treating periodontal disease. This system focuses on administering the medication directly to the base of the periodontal pocket, sustaining the minimum inhibitory concentration, and ensuring that the drug retains its efficacy sufficiently [74]. Using extruded ethylene vinyl acetate fibers infused with 25% tetracycline hydrochloride, Goodson et al. demonstrated tetracycline as a localized drug delivery agent placed and maintained in periodontal pockets [75]. This drug delivery system has been assessed using various medications, including tetracycline, metronidazole, chlorhexidine, minocycline, doxycycline, and chitosan, presented in multiple forms such as fibers, microparticles, strips, films, gels, membranes, ointments, and nanosystems, among others [26,76,77]. Localized drug delivery in periodontics is recommended for specific periodontal pockets that have a probing depth (PD) exceeding 5mm following practical Phase-I therapy, as an adjunct to mechanical debridement, for patients with medical conditions where surgery is not helpful, and in cases of recurrent or refractory periodontitis [78].

Metronidazole

Metronidazole is a specific antibiotic effective against anaerobic gram-negative bacteria [79-81]. The FDA has approved this medication for the treatment of bacterial infections caused by species such as *Prevotella*, *Porphyromonas*, *Bacteroides*, *Fusobacterium*, and *Helicobacter*, as well as protozoal infections like trichomoniasis and microaerophilic infections [33,38,81-83]. Metronidazole comes in many forms, such as topical, vaginal, intravenous, and oral [33,80,82,84], and it exhibits bactericidal activity against anaerobic microorganisms [85]. Metronidazole is a 5-nitroimidazole synthetic drug [86,87]. It functions as a prodrug, requiring metabolic activation by strict anaerobic microorganisms to become effective [39,88-91]. It is also a cost-effective drug and is considered the “gold-standard” drug against the anaerobic activity of microorganisms [92]. For patients allergic to penicillin and its derivatives, metronidazole serves as an ideal drug for the treatment of periodontitis [93]. 

Mechanism of Action (MOA) of Metronidazole

As described in Table 1, the MOA involves a four-step process against anaerobes [94].

**Table 1:** Details the mechanism of action of metronidazole. Credit: Utsav Gandhi.

Steps	Process
1: Entry	Metronidazole enters microorganisms by permeating both aerobic and anaerobic pathogens' cell membranes [95].
2: Activation	By altering the structure of pyruvate-ferredoxin oxidoreductase and creating a concentration gradient, intracellular transport proteins activate metronidazole, improving drug absorption and producing cytotoxic free radicals [96].
3: DNA interaction	It involves the interaction of bacterial DNA with cytotoxic free radicals, causing strand breakage and destabilizing the DNA helix [97,98].
4: Cell death	DNA damage inhibits nucleic acid synthesis and leads to cell death.

Adverse Drug Reactions of Metronidazole

A study done by Kenji et al. in 2014 reported evidence of adverse reactions, including nausea without vomiting (9.9%), nausea with vomiting (1.8%), dysgeusia (1.8%), diarrhea (0.9%), numbness (0.9%), dizziness (0.9%), headache (0.9%), exanthema (0.9%), discomfort (0.9%) [99]. So, it is widely accepted as a well-tolerated drug, but in some rare cases, adverse effects may include peripheral neuropathy, encephalopathy, and optic neuropathy [100]. A reversible metronidazole-induced encephalopathy [101] and neurotoxicity [102,103] is rarely seen. There is insufficient evidence to conclusively establish the genotoxic effects seen in animal studies in humans [97].

The systemic administration of metronidazole in tablet form is prescribed at 250 mg or 500 mg to be taken orally. Through the intravenous route, the dose is 5 mg/mL [33]. Systemic metronidazole may lead to gastrointestinal intolerance, nausea, dysgeusia, headaches, diarrhea, alcohol intolerance, and peripheral neuropathy as its side effects [49,104]. 

Metronidazole as a Local Drug Delivery Agent

**Figure 4:**
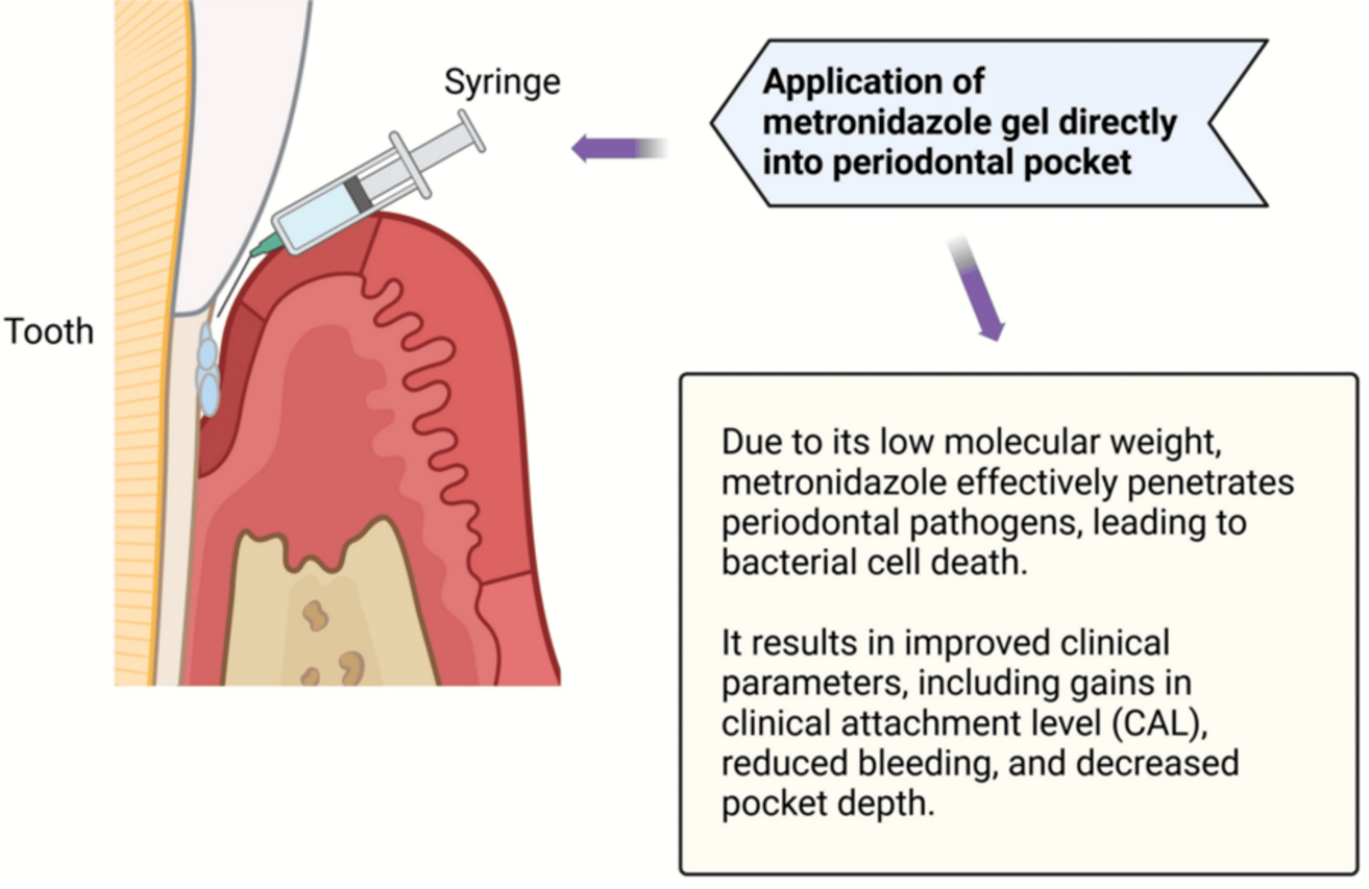
Application of metronidazole as a local drug delivery agent. Notes: The figure was drawn using the premium version of Biorender (https://biorender.com/o22x376) with agreement license number ZW27Z3VU0M [15]. Credit: Utsav Gandhi

Figure 4 displays the administration of metronidazole to the periodontal pocket. Research conducted by Kesarwani et al. in 2022 demonstrated that applying 1% metronidazole gel enhances clinical and microbiological parameters [105,106]. A gel containing metronidazole in 25% concentration is used widely to treat periodontal infection caused by bacterial species [107-111]. It is used to improve clinical measurements such as clinical attachment level (CAL), bleeding during probing (BOP), and PD [112-114]. Using a syringe and a cannula, this fluid is injected into the periodontal pocket [31].

**Figure 5:**
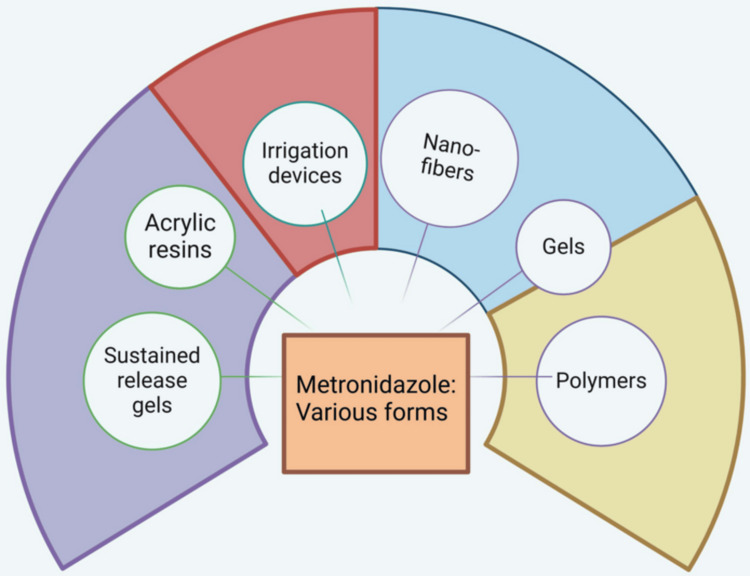
Schematic diagram showing various forms of metronidazole as a local drug delivery agent. Notes: With an agreement license number MY27YBZVPT, the figure was created using Biorender's premium edition (https://biorender.com/s00h712) [15]. Credit: Utsav Gandhi.

Locally administered metronidazole is available in various forms (Figure 5).

Studies Involving Metronidazole As LDD Agent

Table 2 describes the review of clinical studies conducted between 2000 and 2025 on the local administration of metronidazole.

**Table 2:** Clinical studies conducted between 2000 and 2025 on metronidazole. Notes: SRP: Scaling and root planning; Mtz: Metronidazole; MF: Metronidazole; MO: Metronidazole; CAL: Clinical attachment level; BOP: Bleeding on probing; PPD: Periodontal pocket depth; PLGA: Poly (D-L) lactide-co-glycolide; PCL: Poly ε-caprolactone. Credit: Utsav Gandhi.

Studies	Study design	Sample size	Methodology	System	Findings
Nitin Dhegade et al. 2020 [115]	Animal study; study design is not mentioned	Not described	A modified solvent casting process was used to create the intrapocket dental film.	Intrapocket film	Periodontal ligament degeneration is successfully prevented by an initial burst release followed by a sustained drug release for more than 11 days.
Dorota et al. 2019 [116]	A Clinical Pilot Study	n=23	Test group: SRP + with metronidazole-loaded porous matrices, Control group: SRP only	Matrices	Intra-pocket metronidazole in the designed matrix is a valuable addition to conventional periodontal treatment and an alternative to systemic antibiotics.
Miani et al. 2011 [110]	A Randomized Controlled Trial	n=20	Group A: SRP Group B: SRP + gel	Metronidazole containing gel	The gel reduced the bacterial count, while periodontal pathogenic species showed no significant changes.
Bergamaschi et al. 2016 [117]	A Clinical Pilot Study	n=30	Group (i): 3g placebo gel Group (ii): 3g 15% Mtz benzoate gel Group (iii): 3g Mtz (Flagyl(®)) + periodontal debridement	Gel	Metronidazole, whether in gel or tablet form, combined with debridement, demonstrated comparable advantages to placebo in smokers suffering from chronic periodontitis over six months.
Pundir et al. 2021 [106]	A Randomized Controlled Study	n=40	Experimental site – SRP+ 1% MF gel Control site – SRP + placebo	Gel 1%	Applying 1% MF gel improved PPD reduction and CAL gain compared to the placebo gel used as a scaling and root planing adjunct.
Mirzaeei et al. 2021 [118]	Not mentioned	Not described	PLGA and PCL nanofibers were prepared using an electro-spinner	Nanofiber	Nanofibers were released over 7–10 days, with prolonged-release profiles potentially enhancing patient compliance by reducing dosing frequency.
Yang et al. 2001 [119]	A Randomised Clinical Trial	n = 13	Experimental – SRP+ MO gel Standard – SRP+ metronidazole stitus Control – SRP+ placebo	Gel form	The metronidazole gel and metronidazole status groups demonstrated superior clinical outcomes compared to the control group,
Hasan et al. 2020 [120]	A Randomised Clinical Trial	n=30	Group I- Conventional Group II- with Metronidazole gel Group III- with mouthwash	Gel and mouthwash	The gel demonstrated greater efficacy in diminishing clinical attachment loss and inflammatory biomarkers.
Mei et al. 2017 [121]	Animal models	n=10	A solution-gel-based LLC system - for delivering metronidazole into periodontal pockets.	A solution gel form	The LLC system maintained metronidazole levels in periodontal pockets that exceeded the minimum inhibitory concentration for over 10 days.
Toskić-Radojičić, 2005 [122]	A Randomized Controlled Trial	n=25	Experimental site – metronidazole gel, Control site- without treatment	25% metronidazole lipogel	The metronidazole-containing lipogel effectively eliminated anaerobic strains from periodontal pockets within 30 days.
Singh et al. 2016 [123]	A Single-blind, randomized, parallel group clinical study	n=120	Group A: SRP+ Metronidazole Group B: SRP+ Tetracycline Group C: SRP only	Collagen sponge impregnated with 5% metronidazole	Applying local metronidazole with mechanical debridement resulted in a notable reduction of pathogenic flora and a corresponding increase in beneficial microbial flora.
Pandit et al. 2013 [124]	A Randomised Clinical Trial	n=60	Group A: SRP+ minocycline microspheres Group B: SRP+ Metronidazole gel Group C: SRP only	Gel	When compared to SRP alone, both show improvements in PPD and CAL in patients with periodontitis.
Mehravani et al. 2024 [16]	A Randomised Clinical Trial	n=30	Group A: Only SRP Group B: SRP+ Metronidazole tablets Group C: SRP + Metronidazole gel	Gel form	Average CAL improvement at metronidazole gel sites, average BOP decrease in the SRP group, average PPD decrease in tablet and gel form.

Metronidazole's efficacy as a local drug delivery agent for treating periodontal disease has been demonstrated. Its success in this role relies on its therapeutic effectiveness and the delivery systems employed for its administration. The reason behind insufficient therapeutic efficiency is the rapid clearance of drugs from pockets due to salivary flow and its limited durability [125]. Vehicles have been biocompatible, with no toxic effects on the body.

Peri-implantitis is a condition that is both persistent and irreversible, affecting both the hard and soft tissues surrounding an implant, resulting in bone loss [126], reduced osseointegration, more profound pocket formation, and the presence of pus [127-129]. It is treated via surgical and non-surgical options [130]. In certain instances, surgical intervention alone may not sufficiently decrease the presence of pathogenic microorganisms surrounding dental implants [131]. Therefore, systemic or local antibiotics are implemented to achieve superior outcomes [132,133]. Liñares et al. conducted a study demonstrating that administering systemic metronidazole and other non-surgical interventions decreased PD and reduced radiographic defects [132].

To summarize this review, using metronidazole in an intrapocket application is advantageous for enhancing periodontal parameters and serves as a treatment alongside scaling and root planing. It presents as a viable alternative to systemic antibiotics in managing periodontal diseases. 

Limitations of the study

The research requires assessment over an extended period with a more substantial sample size. Clinical and microbiological investigations and systematic reviews are essential for a more comprehensive understanding of metronidazole's role in periodontal disease. Furthermore, the connections between systemic and periodontal diseases warrant further exploration to elucidate their interrelations and potential effects on overall health.

## Conclusions

Metronidazole is an antibiotic commonly utilized in the management of anaerobic infections. An imbalance of microorganisms within the oral cavity can result in dysbiosis and an excessive proliferation of gram-negative anaerobes, which may contribute to periodontal disease. This condition may result in the formation of deeper periodontal pockets, an increase in bleeding, and clinical attachment loss. Metronidazole is a critical component of managing periodontal diseases, acting as an auxiliary to scaling and root planing.
